# The challenge of using patient reported outcome measures in clinical practice: how do we get there?

**DOI:** 10.1186/s41687-024-00711-1

**Published:** 2024-03-21

**Authors:** David Cella, Kyle Nolla, John Devin Peipert

**Affiliations:** Feinberg School of Medicine, Northwestern University, 625 N. Michigan Ave, 2100, Chicago, IL 60611 USA

**Keywords:** Patient-reported outcomes, Clinical decision-making, Meaningful change

## Abstract

**Background:**

As patient-reported outcome measures (PROMs) become available to clinicians for routine clinical decision-making, many wonder how to define a meaningful change in a patient’s PROM score. Some PROMs have a specific threshold that indicates meaningful change, but since those numbers are based on population averages, they do not necessarily apply to the varying experiences of each individual patient. Rather than viewing this as a weakness of PROMs, it is worth considering how clinicians use other existing measures in clinical decision-making—and whether PROMs can be used similarly.

**Body:**

An informal survey of 43 clinicians reported using measures such as weight, blood pressure, and blood chemistry to inform clinical decision-making. Although clinicians were very consistent with what constituted a meaningful change for some measures (e.g., ECOG performance status), other measures had considerable variability (e.g., weight), often informed by their specialization (for example, differing thresholds for meaningful weight change for adult primary care, pediatrics, and oncology). For interpreting change in measures, they relied on clinical experience (44%), published literature (38%), and established guidelines (35%). In open-response comments, many clarified that the results of any measure had to be taken in the context of each individual patient before making treatment decisions. In short, clinicians already apply individualized clinical judgment when interpreting score changes in existing clinical measures. As clinicians gain familiarity with PROMs, PROMs will likely be utilized in the same way.

**Conclusion:**

Like other clinical measures from weight to blood chemistry, change in a PROM score is but one piece of a patient’s clinical story. Rather than relying on a hard-and-fast number for defining clinically meaningful change in a PROM score, providers should—and many already do—consider the full scope of a patient’s experience as they make treatment decisions.

## Background

Several recent trends have led to increased interest in the use of patient reported outcome measures (PROMs) in clinical practice. These include perspectives from the patient advocacy community [1], regulatory agencies that encourage PROM collection to help them weigh risks and benefits of new drugs [2], accrediting agencies and third party payers that endorse healthcare quality measures [3], professional societies [4], and clinicians themselves. When successful, it can lead to improved patient care and contribute to hospital and healthcare delivery organization rankings [5–7]. Despite this, the routine use of PROMs in clinical practice remains rare, often due to practical challenges in implementation. Another more abstract barrier is a certain skepticism from clinicians regarding whether PROM data add value to individual patient management. After all, through interview and consultation, clinicians routinely gather patient input on symptoms, function, and sometimes even general quality of life. Does structured, formal assessment of these aspects of patient health with a PROM improve upon informal assessment? And if so, how are clinicians expected to use these PROM results? These are fair questions.

The PROM community has published thousands of articles demonstrating the reliability, validity, and clinical relevance of an array of PROMs. As members of that PROM community, we are often asked some version of these questions by clinicians integrating PROMs into their practice: What is a clinically meaningful score? What is a meaningful change in score? When does a score or change in score necessitate clinical action for a given patient? While methodologists still debate the exact answers to these questions [8], many PROMs offer published estimates of meaningful difference and change scores that can serve as reference values for careful clinical consideration [9, 10 ]. However, these clinically meaningful change estimates should not be taken as immutable standards, even when the PROM is well-established as an excellent measure [11]. Instead, PROM change scores should be interpreted as a guideline: a meaningful change in PROM score as defined by a published article may or may not indicate a need for change in clinical management of an individual patient [12]. Indeed, studies suggest that individual patients define a meaningful change in PROM score differently, even when they have the same condition—furthermore, patient definitions of meaningful change often differ from clinician definitions [13]. So, a meaningful change in score according to an article or even according to a clinician’s experience may not be a meaningful change in score to a specific patient. Still, we argue that balancing an individual patient’s subjective experiences and unique needs with their clinical measurement results is how medicine is practiced with virtually every clinical measure in use today—not just with PROMs.

## How clinicians use change scores

To illustrate how clinicians use their judgment to interpret the clinical relevance of change, whether in biomarkers or PROMs, we conducted a brief email query. We emailed an anonymized free-response survey to clinical colleagues throughout the US and asked them to identify up to 5 clinical measures of any kind they use in practice and to tell us the basis (justification) for a meaningful change in that measure. We also asked the specific value that would denote a meaningful change so that we could compare values across respondents who entered the same clinical measure. We invited 130 colleagues to educate us; 43 agreed. Two of the colleagues who declined wrote a reply email saying the request over-simplified the use of clinical measures in practice. Most respondents (32) were men, with average age 59 years (range 35–72), and 27 years of experience (range 3–46). Specialties included oncology (14), surgery (6), rheumatology (3), dermatology (2), internal medicine (2), gynecology (2), and neurology (2). These 43 colleagues reported 156 measures that they use, with three common justifications: they determined whether a change in the measure was clinically meaningful based on clinical experience (44%, 69/156), published research (38%, 59/156), and established guidelines (35%, 54/156). Respondents submitted over 100 unique measures; a few were submitted by multiple clinicians: ECOG performance status (8), weight (8), pain (7), hemoglobin (7) and blood pressure (6). For some of these measures, clinicians had very high agreement about what constituted a meaningful change. For example, nearly everyone agreed that a change of one level of ECOG performance status was clinically significant. This stands to reason, since the ECOG performance status measure has discrete categories representing large gaps in symptom experience and ambulation. However, for other measures like weight, hemoglobin, and blood pressure, responses for what constituted a meaningful change were diverse, which likely reflects the unique clinical contexts and patient populations that these doctors work with. For example, the specific value of meaningful weight loss differed in adult primary care, pediatrics, and oncology—as it should for these very different contexts.

The comments we received were telling, often reflecting the importance of weighing other considerations when interpreting change. For example: “I believe trying to establish a meaningful change/difference for a given measure is meaningless because any change, even an extremely small one, can be important”; “For some clinical measures it is hard to say what determines a clinical meaningful change”; “How meaningful a change is in a specific clinical measure often requires consideration of the full clinical context”; “there is a lot of variation within the same clinical measure”; “there is a feel to these numbers with variability in interpretation (judgment) depending on the specific patient and their broader health circumstances”; “None of my meaningful change thresholds are absolutely compelling. All are rules of thumb and the ultimate interpretation still requires the full clinical context of the individual patient’s pattern of symptoms and experience of their health and illness”; and finally: “Why are questionnaires held to much higher standards in defining [minimal clinically important difference] relative to ‘physical’ measures?”.

Indeed, this last comment gets right to the point. Our informal, arguably unscientific email exchange illustrated what is obvious to many: Clinical measures in common use, such as weight, blood pressure, and blood chemistry values, are best applied to individual patient care with some flexibility and nuance, even when they are based on practice guidelines or published literature. As is the case with these clinical measures, PROMs should not be used rigidly, but rather incorporated into practice with other clinical and personal data. For clinicians who are already using PROMs, interpretation typically involves a simple comparison between earlier and recent scores, but many clinicians also report considering a patient’s other conditions in their judgments, as well as population norms for the PROM [14]. We encourage colleagues to study clinical use of PROs more formally in the future.

## Conclusions

How do we get to the point that PROMs are treated with the same flexibility and confidence as other clinical measures? We suggest the answer comes from familiarity and use in practice. Just as with common clinical measures such as weight, performance status, blood pressure, blood chemistry, and pain, the amount of change that is meaningful for one person may not be for another, and the relevance of that change to necessary clinical action often depends on factors beyond the value of the score change. We also encourage PRO methodologists to recognize that a range of values, rather than a single value, may reflect meaningful change at the patient level because range is more inclusive of unique individuals and contexts. PROMs, like many other clinical measures, are best used in the hands of a knowledgeable and experienced clinicians. The only difference with PROMs is their subjectivity, as they draw from the rich and varied perspective of the individual patient. In that regard, their use requires close collaboration with that individual patient. Then again, the same could be said about all clinical measures.

Bottom line: Find a measure that is relevant to your clinical area and use it routinely. Discuss with your patients and discover how their self-report can augment other clinical data. Over time you will become proficient and discover when and how to add formal PROM assessment into clinical practice. With time, experience will make the measure meaningful.

## Data Availability

Available upon reasonable request to the corresponding author.

## List of abbreviations

PROM: Patient-reported outcome measures
ECOG: Eastern cooperative oncology group
